# Identification and characterization of EBV genomes in spontaneously immortalized human peripheral blood B lymphocytes by NGS technology

**DOI:** 10.1186/1471-2164-14-804

**Published:** 2013-11-19

**Authors:** Haiyan Lei, Tianwei Li, Guo-Chiuan Hung, Bingjie Li, Shien Tsai, Shyh-Ching Lo

**Affiliations:** 1 Tissue Microbiology Laboratory, Division of Cellular and Gene Therapies, Office of Cellular, Tissue and Gene Therapy, Center for Biologics Evaluation and Research, Food and Drug Administration, NIH Building 29B, Rm. 1NN06, 29 Lincoln Dr., 20892-4555 Bethesda, MD, USA

**Keywords:** Epstein Barr Virus, Mycoplasma, Next-generation sequencing, Human immortalized B lymphocytes

## Abstract

**Background:**

We conducted genomic sequencing to identify Epstein Barr Virus (EBV) genomes in 2 human peripheral blood B lymphocytes that underwent spontaneous immortalization promoted by mycoplasma infections in culture, using the high-throughput sequencing (HTS) Illumina MiSeq platform. The purpose of this study was to examine if rapid detection and characterization of a viral agent could be effectively achieved by HTS using a platform that has become readily available in general biology laboratories.

**Results:**

Raw read sequences, averaging 175 bps in length, were mapped with DNA databases of human, bacteria, fungi and virus genomes using the CLC Genomics Workbench bioinformatics tool. Overall 37,757 out of 49,520,834 total reads in one lymphocyte line (# K4413-Mi) and 28,178 out of 45,335,960 reads in the other lymphocyte line (# K4123-Mi) were identified as EBV sequences. The two EBV genomes with estimated 35.22-fold and 31.06-fold sequence coverage respectively, designated K4413-Mi EBV and K4123-Mi EBV (GenBank accession number KC440852 and KC440851 respectively), are characteristic of type-1 EBV.

**Conclusions:**

Sequence comparison and phylogenetic analysis among K4413-Mi EBV, K4123-Mi EBV and the EBV genomes previously reported to GenBank as well as the NA12878 EBV genome assembled from database of the 1000 Genome Project showed that these 2 EBVs are most closely related to B95-8, an EBV previously isolated from a patient with infectious mononucleosis and WT-EBV. They are less similar to EBVs associated with nasopharyngeal carcinoma (NPC) from Hong Kong and China as well as the Akata strain of a case of Burkitt’s lymphoma from Japan. They are most different from type 2 EBV found in Western African Burkitt’s lymphoma.

## Background

Epstein-Barr virus (EBV) is a ubiquitous gamma-herpesvirus highly prevalent in all human populations. The virus is associated with both non-malignant diseases and a number of human cancers. After the first discovery of EBV particles in cultured lymphoma cells from patients with Burkitt’s lymphoma (BL) [1,2], EBV and its encoded molecules were also detected in other disease processes and malignancies such as undifferentiated nasopharyngeal carcinoma (NPC) [3], infectious mononucleosis [4], Hodgkin’s disease [5,6], and gastric carcinoma [7,8] etc.

Eight EBV whole-genome sequences have been reported from previous studies. B95-8 (GenBank accession number V01555.2), derived from a North American case of infectious mononucleosis [9], was the first completely sequenced EBV genome. A more complete 171 kb wild-type EBV reference genome, WT-EBV, (GenBank accession number NC_007605.1/AJ507799.2) was later constructed using B95-8 as a backbone while an 12 kb missing fragment in the EBV genome was provided by the EBV sequence found in Raji cells [10,11]. AG876 (DQ279927.1) from a Western African case of Burkitt’s lymphoma is the only complete type 2 EBV sequence to date [12]; GD1 (AY961628.3), GD2 (HQ020558.1) and HKNPC1 (JQ009376.1) are all EBV genomes derived from NPC patients or NPC tumours. Specifically, GD1 was isolated from the saliva of an NPC patient, while GD2 and HKNPC1 were isolated from an NPC tumour or tumour biopsy. GD1 was sequenced using the conventional shotgun sequencing. GD2 and HKNPC1 were very recently sequenced using the next generation sequencing (NGS) Illumina platform [13-15]. Moreover, 2 EBV genomes, Akata (KC207813) and Mutu (KC207814), were reported recently from cases of Burkitt’s lymphomas from Japan and Kenya, respectively, by NGS technology [16].

Our laboratory has recently established a new high throughput sequencing (HTS) capability using the MiSeq platform. Currently, we are evaluating various workflows using different programs/tools for the effective detection of a target viral genome as well as novel viral genes in computational analysis of massive amounts of sequencing data generated by NGS technology. Our objective is to effectively detect and characterize infectious viruses in human host cells or tissues intended as therapeutic biologics by using the new technology. For this purpose, we conducted an experimental genomic sequencing study of 2 human B cell lines that underwent spontaneous immortalization promoted by infection of the cell culture with mycoplasma [17]. These 2 immortalized human B lymphocyte cell lines were previously shown to be monoclonal in nature and positive for the EBV LMP1 antigen [17]. Using available computational tools, we identified and extracted EBV sequences from genomic sequencing data of the 2 B-lymphocyte cell lines, assembled them into single genomes and compared them with the sequences of all the EBV genomes previously reported to the GenBank. In addition, we identified and extracted EBV sequences from the 1000 human genomes project sequences which had been deposited in NCBI for comparison. The project has been studying human genomic DNA of human peripheral blood B lymphocytes immortalized by EBV infections in culture. The present study provided a rapid genomic comparison and characterization of EBV strains associated with different human diseases along with their pathogenesis and geographical distributions.

## Results

### Taxonomic classification of sequences obtained from MiSeq

**Figure 1:**
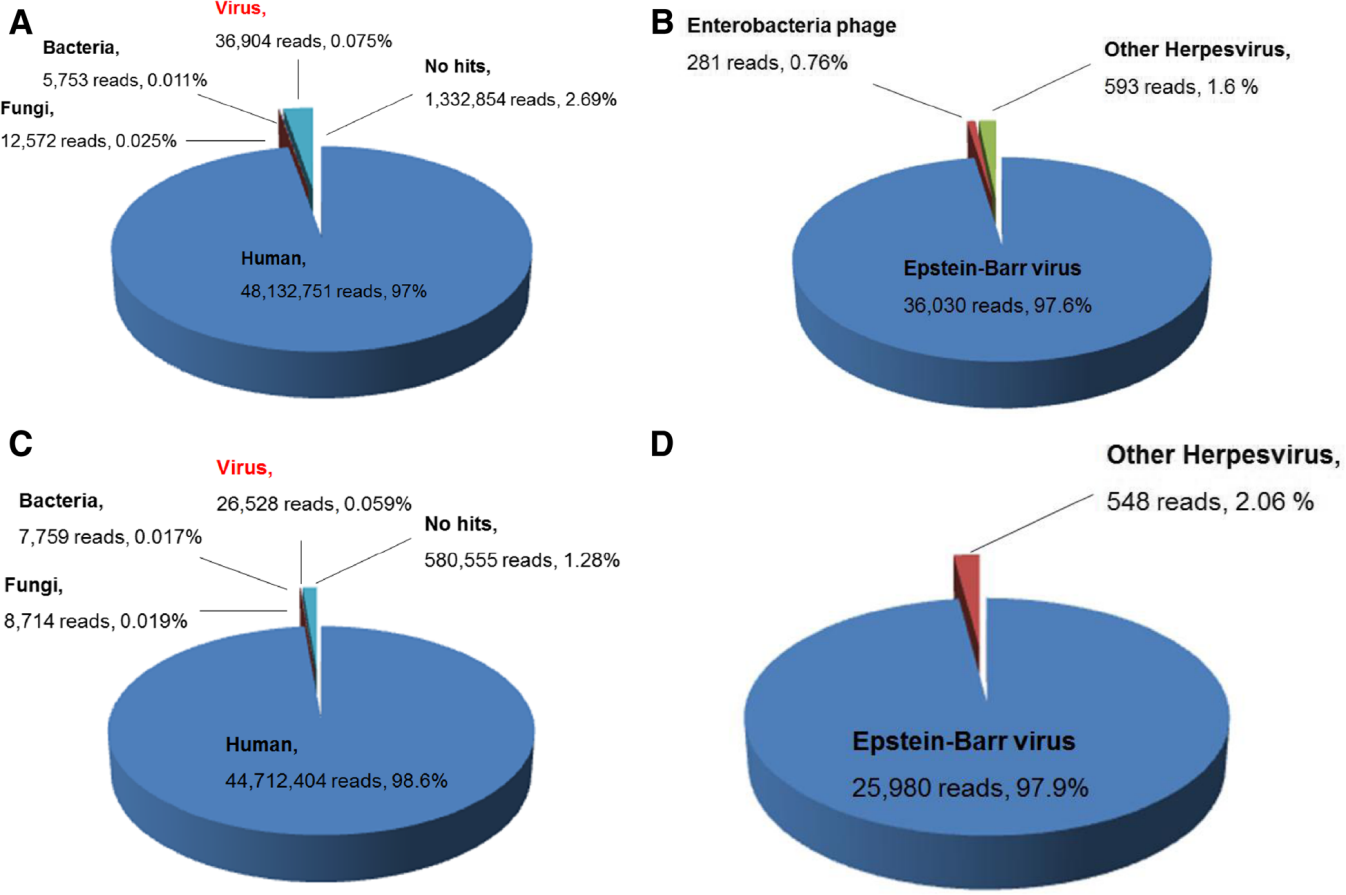
**Summary of the distribution of K4413-Mi and K4123-Mi sequence reads mapping to human, bacterial, fungal, and viral databases.** The pie charts were generated based on the results of a homology search for all of the short reads. **(A)** K4413-Mi all reads distribution. **(B)** K4413-Mi virus reads distribution. **(C)** K4123-Mi all reads distribution. **(D)** K4123-Mi virus reads distribution.

DNAs were isolated from the cultures of 2 previously established, spontaneously immortalized human B lymphocyte cell lines (K4413-Mi and K4123-Mi) for genomic sequencing. A total of 49,520,834 sequencing reads of 168-base paired-end reads were generated by the MiSeq sequencer resulting in 8.3 Gb of sequence data from the K4413-Mi cell line. Similarly, a total of 45,335,960 reads of 198-base paired-end reads resulting in 8.9 Gb of sequence data were collected from the K4123-Mi cell line. Those short reads that passed quality control filtering were first aligned to the database of human reference genome sequences (hg19, GRCh37; http://hgdownload.cse.ucsc.edu/goldenPath/hg19/bigZips/). After removing the sequencing reads that aligned with human reference genome database, the remaining reads were aligned against the bacterial, fungal, and viral nucleotide databases sequentially (ftp://ftp.ncbi.nlm.nih.gov/genomes/Bacteria/, 04/27/2013 version; ftp://ftp.ncbi.nlm.nih.gov/genomes/Fungi/, 03/25/2013 version; ftp://ftp.ncbi.nlm.nih.gov/genomes/Viruses/, 05/22/2013 version) to determine the sequence composition using CLC Genomic Workbench (http://www.clcbio.com/, version 6.0.2). Of the 49,520,834 sequencing reads from K4413-Mi cell line, 97.2% (48,132,751 reads), 0.011% (5,753 reads), 0.025% (12,572 reads), and 0.075% (36,904 reads) were classified as human, bacteria, fungi, and virus related sequences, respectively (Figure 1A). Of the 45,335,960 original reads from K4123-Mi cell line, 98.6% (44,712,404 reads), 0.017% (7,759 reads), 0.019% (8,714 reads) and 0.059% (26,528 reads) were classified as human, bacteria, fungi, and virus related sequences, respectively (Figure 1C).

Of the virus-related reads identified in both cell lines, 97.6% of 36,904 reads in the K4413-Mi cell line and 98.1% of 26,528 reads in the K4123-Mi cell line were classified as EBV-specific sequences (Figure 1B, 1D). In the K4413-Mi cell line virus-related reads, there were 281 short reads classified as sequences related to *Enterobacteria* phage phiX174. There were 593 and 548 short reads classified as “other Herpesvirus” in K4413-Mi and K4123-Mi cell line virus-related reads, respectively (Figures 1B, D).

### Assembly of EBV genomes from sequences mapping with WT-EBV

In order to assemble the K4413-Mi and K4123-Mi EBV genomes, we mapped the total sequencing reads of K4413-Mi and K4123-Mi to the reported EBV reference genome (WT-EBV, NC_007605) separately using the CLC Genomics Workbench. The mapped reads were considered to represent EBV-related sequences. Overall 37,757 out of 49,520,834 total reads in one lymphocyte line (K4413-Mi Line) and 28,178 out of 45,335,960 total reads in the second lymphocyte line (K4123-Mi Line) were identified as EBV-related sequences. The EBV-related sequences identified in K4413-Mi cell line covered 99.99% of the entire reference WT-EBV genome. The average coverage was 35.22-fold. There were 10 gaps in the K4413-Mi EBV genome assembled, with gap lengths varying from 1 bp to 105 bp. The total gap length was 352 bp. For the K4123-Mi EBV genome assembly, the EBV-related sequences identified covered 99% of the WT-EBV reference genome. The average coverage was 31.06-fold. There were 18 gaps in the K4123-Mi EBV genome assembled, and the gap length varied from 1 bp to 177 bp. The total gap length was 993 bp. Finally, the complete genome of K4413-Mi EBV was 171,843 bp (with 352 “N”). The K4123-Mi EBV genome was 171,793 bp (with 993 “N”). Both genomes have a GC-content of 59.5%. Both genomes were also annotated using the information derived from the reference genome of WT-EBV (NC_007605) sequence, and submitted to GenBank (GenBank accession number KC440852 and KC440851).

### Identification and extraction of EBV sequences from NA12878 data

To evaluate if the bioinformatics tool used in the study could be equally effective in identifying and characterizing viral genome sequences from much more massive human genome sequencing data generated by NGS technology and to compare genome sequences of EBV identified in our “spontaneously” immortalized B cells with those in B cells transformed by acute EBV infection in culture, we downloaded sequencing raw data from NCBI of 1000 human genomes project. In the human1000 genomes project (http://www.1000genomes.org/), human genomic DNA was isolated from human B cells immortalized by EBV infection in culture [18] and deeply sequenced. The human genomic sequences obtained by this project have been deposited in NCBI. In this study we identified and extracted the EBV sequences from NA12878 human genomic sequences deposited in NCBI for comparison with EBVs in our B-cell lines. We downloaded 846,783,709 Illumina 36 bp short reads from ftp://ftp-trace.ncbi.nih.gov/1000genomes/ftp/data/NA12878/sequence_read/ and used CLC Genomics Workbench software to map these reads with the WT-EBV reference genome (NC_007605). In the total reads downloaded, 2,219,104 (0.26%) short reads mapped with the WT-EBV and covered 100% of the reference genome. We assembled the NA12878 EBV genome sequence from the raw reads mapping to the WT-EBV. We obtained a single contig with 171,852 bp consensus sequence from the assembly. We named the consensus sequence of the EBV genome NA12878 EBV. It was estimated that each immortalized human lymphocyte induced by infection of B95-8 EBV carried 102 copies of EBV genomes.

**Figure 2:**
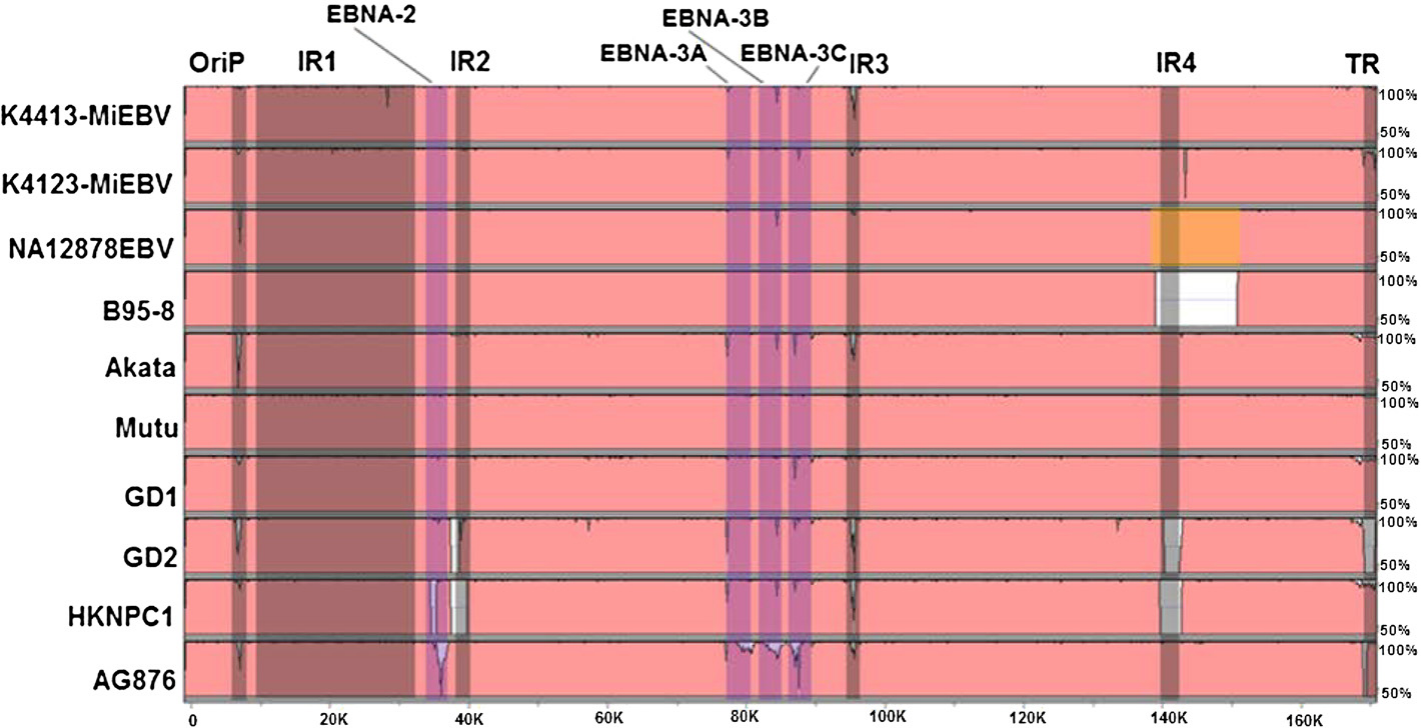
**Pair-wise alignment of genomic sequences for the newly assembled K4413-Mi EBV, K4123-Mi EBV, NA12878 EBV, and other 7 EBV strains previously reported to GenBank with the WT-EBV genome.** The 10 EBV genomes examined generally have high sequence identity with WT-EBV, except repeat regions (brown) and polymorphic genes (purple). EBNA3A, -3B, and -3C genes show lower sequence identity in AG876 (type 2). The region marked with yellow colour in the NA12878 EBV genome has a significantly lower coverage than the rest of NA12878 EBV genome in genome assembly. The figure was generated by mVISTA software, using 100-bp moving window with minimum identity of 50% and maximum identity of 100%.

Since the large majority of the human peripheral blood B lymphocytes used in the human genomes study were transformed in culture by infections of B95-8 strain of EBV, it was surprising that the segment of sequence (~ 12 kb) between 139 kb and 151 kb of WT-EBV genome that B95-8 EBV genome does not possess could also be found in the assembled NA12878 EBV consensus genome sequence. Further analysis however revealed this specific ~ 12 kb segment of gene sequences (marked with different colour on NA12878 EBV consensus genome in Figure 2) was being covered less than 1/20 folds compared with the average 464.9-fold coverage of the rest of EBV genome. It appeared a small portion of human peripheral blood B cells transformed by B95-8 strain of EBV in 1000 human genomes project might already be infected by EBVs carrying the gene segment that was not present in B95-8 EBV. But the sequencing data clearly demonstrated the large majority of EBV genome sequences deposited in the project appeared to be closely related to the B95-B strain lacking this specific segment of genes.

### K4413-Mi EBV and K4123-Mi EBV are more closely related to B95-8, WT-EBV, Mutu and NA12878 EBV

Pair-wise alignment of the newly assembled K4413-Mi EBV, K4123-Mi EBV, NA12878 EBV and 7 other EBV strains previously reported to GenBank with the WT-EBV genomic sequence was performed. Figure 2 shows the comparison result displayed by using the Mvista program (http://genome.lbl.gov/vista/mvista/submit.shtml). Overall sequence similarities for the WT-EBV genome with the 10 other EBV genomes are high: K4413-Mi EBV (99%), K4123-Mi EBV (99%), NA12878 EBV (100%), B95-8 (95%), Akata (98%), Mutu (100%), GD1 (97%), GD2 (96%), HKNPC (96%), AG876 (96%). The region (13 kb - 42 kb) with highly repetitive sequence in the EBV genome where majority of gaps and ambiguity reads occurred was not included for the genomic comparison (Figure 2). Figure 2 shows that sequences in OriP (origin of DNA replication), IR 3, 4 (internal repetitive family), and TR (terminal repetitive family) regions among the EBV genomes have lower similarity. There were also many sequence variations in polymorphic genes, such as EBNA-3A, 3B, and 3C among these EBV genomes (Figure 2).

More importantly, comparison of sequences in the region spanning EBNA3A, 3B and 3C for K4413-Mi EBV and K4123-Mi EBV against WT-EBV genome revealed much higher similarity with those of the B95-8 and Mutu genomes, but very different from the sequence in this region of the AG876 genome.

### Phylogenetic comparison for the genomic sequences of K4413-Mi and K4123-Mi EBV with other EBVs associated with various human diseases and from different geographic areas

**Figure 3:**
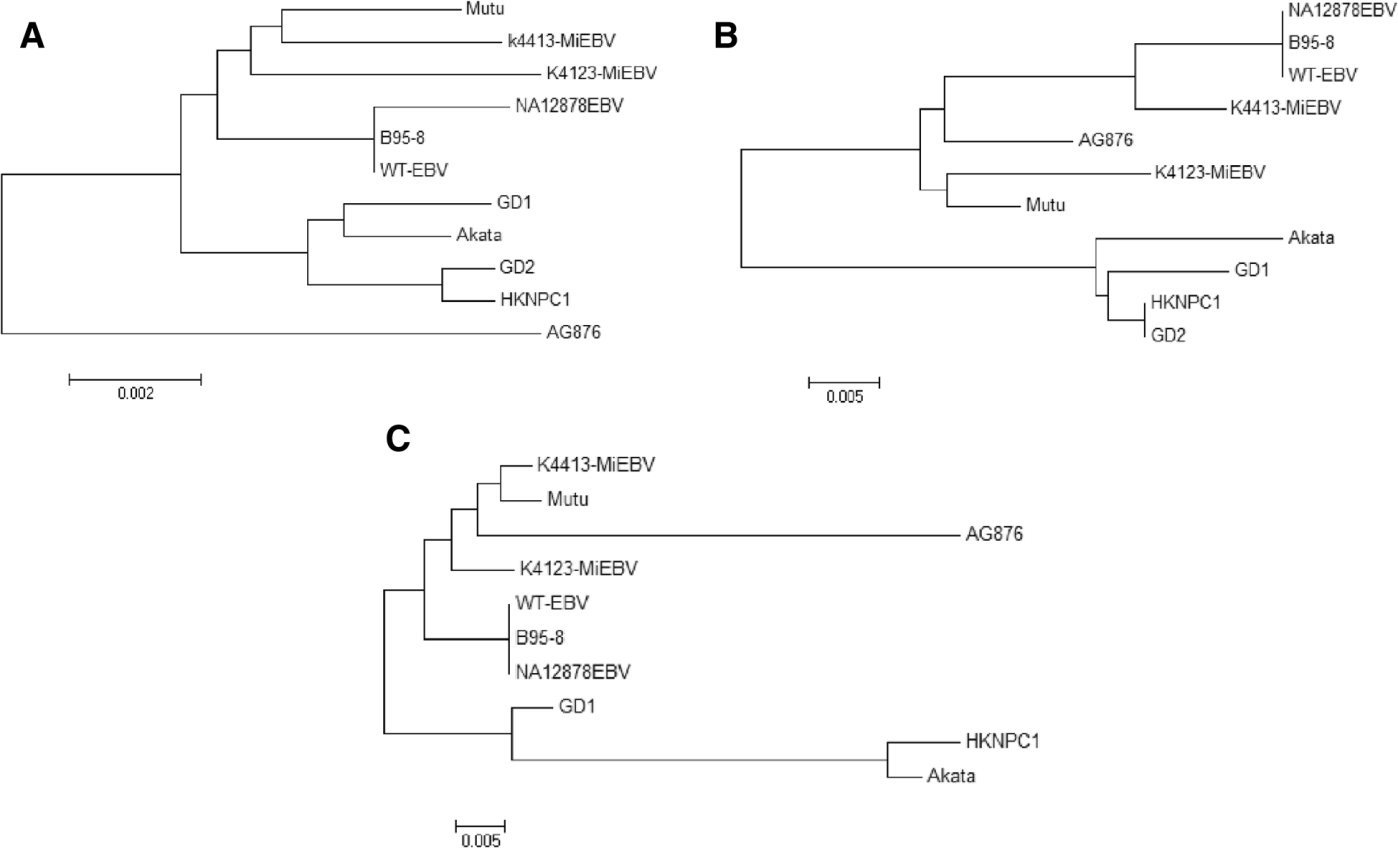
**Phylogenetic analysis of the 11 EBV genomes based on variations in the alignment of DNA and protein sequences of the 11 EBV strains.** Phylogenetic trees based on **(A)** DNA sequence alignment of the 11 EBV genomes. **(B)** Protein sequence alignment of LMP-1. **(C)** Protein sequence alignment of EBNA-1. GD2 EBNA-1 is not included because the original GD2 EBNA-1 protein sequence is not available. Alignment was done with the Kalign (for DNA) and Clustal Omega (for protein) program in EBI; Phylogenetic analysis was performed using MEGA software (version 5), by Neighbour-joining (NJ) algorithm. The divergence scale (showing numbers of substitutions per site) is indicated at the foot of each tree.

Multiple sequence alignment of K4413-Mi EBV, K4123-Mi EBV, and the other 7 EBV genome sequences was performed using the Kalign program in EBI (http://www.ebi.ac.uk/Tools/msa/kalign/) and the Neighbour-joining trees were constructed using MEGA 5 software (http://www.megasoftware.net/) (Figure 3A). Phylogenetic analysis revealed that K4413-Mi EBV and K4123-Mi EBV were more closely related to B95-8, WT-EBV and NA12878 EBV. They were more different from GD1, GD2, and HKNPC1, EBVs associated with NPC found in China and Hong Kong as well as Akata, EBV from a case of Burkitt’s lymphoma found in Japan. They were most different from AG876, the type 2 EBV found in a case of the Western African Burkitt’s lymphoma. Interestingly, Mutu EBV from a case of Burkitt’s lymphoma in Kenya is found to be more closely related to K4413-Mi EBV, K4123-Mi EBV and B95-8 and WT-EBV (Figure 3A). Phylogenetic trees generated from the alignment of translated amino acid sequences of LMP1 and EBNA-1 also showed that K4413-Mi EBV and K4123-Mi EBV are more distant from the NPC associated EBVs and Akata strain of EBV from Burkitt’s lymphoma in East Asia (Figures 3B, C).

### Identification of variations from 2 immortalized human B cell lines

**Figure 4:**
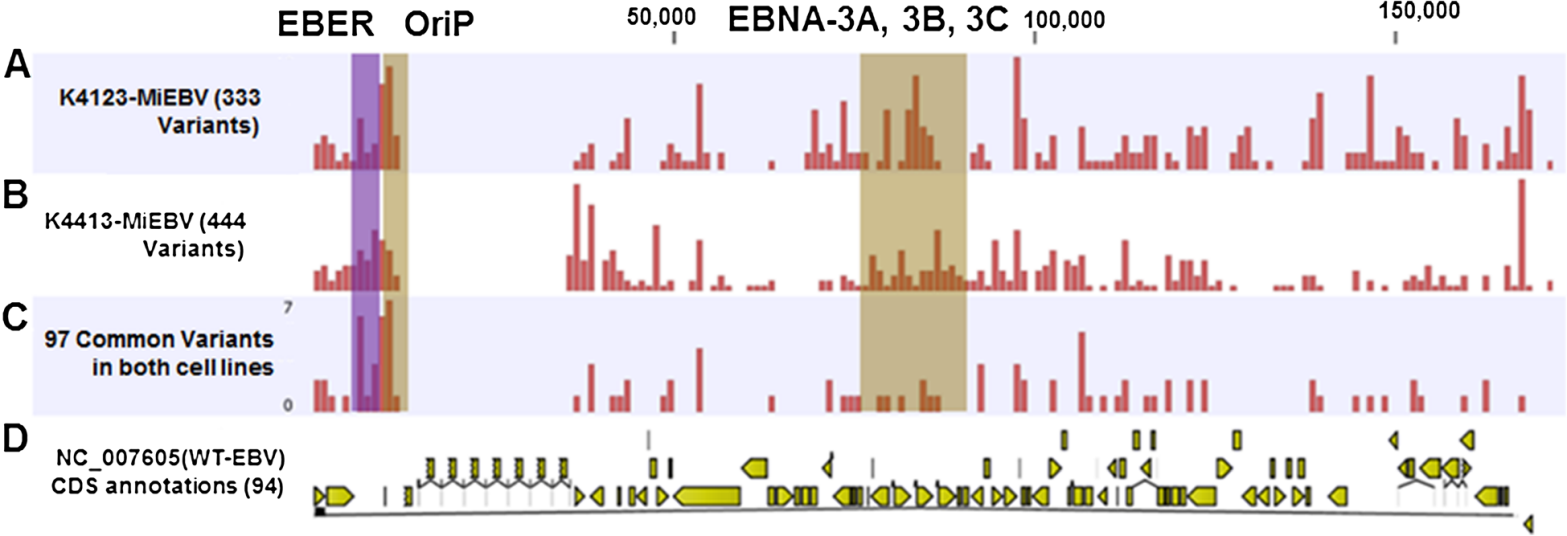
**Variants detected in EBV genomes of the 2 immortalized human B cell lines– in comparison with WT-EBV.** The red bar indicates the number of variants in the 1 kb region. **(A)** 333 variants with genome-wide distribution were found in the K4123-Mi EBV genomes. **(B)** 444 variants with genome-wide distribution were found in the K4413-Mi EBV genomes. **(C)** 97 common variants were found in both K4413-Mi EBV and K4123-Mi EBV genomes. **(D)** The CDS annotations for WT-EBV. The figure was generated by CLC Genomics Workbench.

We used the CLC Genomics Workbench to identify variations, including single-nucleotide variants (SNVs), indels (insertion and deletions) in K4413-Mi and K4123-Mi EBVs. In comparison with the WT-EBV reference genome, a total of 444 variants with genome-wide distribution were found in the K4413-Mi EBV genomes (Figure 4B). Among them, 418 variants were homogenous SNVs; 10 variants were heterogeneous SNVs. In addition, there were 9 insertions and 7 deletions. There were 424 variants located in the coding regions and 138 variants were non-synonymous substitutions (Table 1 and Additional file 1: Table S1). In comparison with the WT-EBV reference genome, a total of 333 variants with similar genome-wide distribution were found in the K4123-Mi EBV genome (Figure 4A). Among them, 324 variants were homogenous SNVs; 1 variant was heterogeneous SNV. In addition, there were 2 insertions and 6 deletions. Among these variants, 329 variants were located in the coding region and 110 variants were non-synonymous substitutions (Table 1 and Additional file 2: Table S2).

**Table 1:** Summary of variations identified in EBVs of the K4413-Mi and K4123-Mi cell lines–in comparison with WT-EBV

	**K4413-Mi EBV**	**K4123-Mi EBV**
**No. of variations**	444	333
**No. of SNVs (single-nucleotide variants)**	428	325
**No. of homozygous SNVs**	418	324
**No. of heterozygous SNVs**	10	1
**No. of indels (insertion or deletion)**	16	8
**No. of coding regions change**	424	329
**No. of non-synonymous substitutions**	138	110

It is important to note that K4413-Mi and K4123-Mi shared 97 common variants (Figure 4C). The common variants were apparently clustered in certain specific regions, e.g., from 6 kb to 10 kb, which is the region that encodes EBER-1, 2 (EBV-encoded RNA) and contains the OriP (the latent cycle origin of DNA replication), and from 79 kb to 83 kb, the region that encodes EBNA3 etc (Figure 4).

## Discussion

We are evaluating the capability of detecting/characterizing infectious viruses in human host cells through HTS genomic sequencing using the Illumina Miseq platform and different computational tools. In this study, we examined 2 human B lymphocyte cell lines that underwent spontaneous immortalization promoted by mycoplasma infection of the cell culture using the Illumina MiSeq platform. We used the HTS data analytic program, CLC Genomics Workbench, to classify the massive short sequence reads (49.5 and 45.3 million reads, ~175 bp/each) generated from genomic sequencing of the 2 cell lines by mapping these sequences with human, bacterial, fungal, and viral genomic databases from NCBI database respectively (Figure 1A-D). The majority of reads obtained (97.2% or 8.1 Gb for K4413-Mi cell line and 98.6% or 8.8 Gb for K4123-Mi cell line), that was classified as human sequences, had approximately 1.22-fold and 1.34-fold coverage of a diploid human genome (6.6 Gb).

Consensus EBV genomes were constructed according to the mapping result of the raw read with WT-EBV by using the CLC Genomics Workbench. The K4413-Mi EBV consensus genome was assembled by using 37,757 EBV-related reads (0.075% of total sequencing raw reads), had 171,843 bp in length with ~ 35.22-fold coverage of EBV genome. Thus, it is estimated that there are ~29 copies (35.22 vs 1.22 coverage) of EBV genome in a single cell of the K4413-Mi lymphocyte cell line. Similarly, the K4123-Mi EBV genome was assembled by using 28,178 EBV-related reads, and was 171,793 bp in length with ~ 31.06-fold coverage of the EBV genome. It is estimated that there are ~23 copies (31.06 vs 1.34 coverage) of the EBV genome in a single cell of the K4123-Mi lymphocyte cell line. The genome copy numbers of EBV found in these resting human B lymphocytes that underwent spontaneous immortalization are apparently higher than those previously reported in the undifferentiated NPC tumours. In GD2, there were ~ 6 copies of EBV genome found in a single NPC tumour cell [14]. In comparison, the NA12878 EBV genome constructed from NCBI database revealed an average of 102 copies of EBV genome in each immortalized human lymphocyte induced by infection of B95-8 EBV in culture. It appears that there are many more copies of EBV genomes that are present in each transformed human B lymphocyte induced by acute B95-8 EBV infection than in spontaneously transformed EBV-positive B-cell promoted by infections of mycoplasma. The copy number of EBV genomes found in an NPC tumour cell is evidently the lowest.

Analysis of our results show that some sequences, albeit very few, obtained in the genomic sequencing of K4413-Mi and K4123-Mi human lymphocyte cell lines mapped to bacteria, fungi, and non-EBV viruses (Figure 1A-D). None-biased parallel HTS is capable of picking up trace amounts of DNA molecules present in culture media and serum. Most of the bacteria-related sequences were mapped to *Enterobacteria*. When matched with the NCBI viral database using the CLC Genomics Workbench, 281 reads in K4413-Mi cell line were found to be *Enterobacteria* phage phiX174. It is possible that the bacteria in the medium also carried the *Enterobacteri*a phage phiX174. Consistent with the original study finding [17], no mycoplasma sequence was identified in these cell lines. There are 593 reads in K4413-Mi cell line and 548 reads in K4123-Mi were classified to be other Herpesvirus-related sequence, not the EBV virus (Figure 1B, D). We conducted the individual BlastN for these 1141 reads. Most of these reads also matched with EBV virus with low homology, and some reads matched with Human sequence. In this context, 2.69% and 1.28% of reads showed “no hits” in mapping against the 4 genomic databases (human, bacteria, fungi, and virus) for the K4413-Mi and K4123-Mi cell lines, respectively. When these sequences were mapped using BlastN against NCBI non-redundant databases, most of these sequences were found to be human sequences. The CLC Genomics Workbench is a very powerful tool for NGS data analysis. It allows us to quickly identify the read composition from the massive amount of data. We also can effectively detect and assemble the target viral EBV genome with the CLC Genomics Workbench. Of course, all software programs have its advantages and drawbacks. For bioinformatics tools, parameter settings and databases used will affect the outcome of the analysis. Different bioinformatics tools are likely to produce different results. We also tried the DNASTAR, SOAP package, BWA and other bioinformatics tools for our analysis. The results are slightly different from using the CLC Genomics Workbench (data not shown). In this study, we only present our analysis with the CLC program.

Whole-genome sequencing of EBV in the infected cells enabled the determination and thus comparison of EBV variations at the genome level. The constructed genomes of K4413-Mi EBV and K4123-Mi EBV are highly similar to each other and to the 8 other reported EBV genomes in the GenBank. However, there are apparent degrees of variations among the genomes of these EBVs studied. Phylogenetic comparison of these EBV genomes revealed that K4413-Mi EBV and K4123-Mi EBV are more closely related to B95-8, EBV isolated from a patient with infectious mononucleosis. They are evidently more distant from GD1, GD2, and HKNPC1, EBVs associated with NPC tumours. Specific comparison for the two particular EBV genes (LMP1 and EBNA-1) that were considered risk-loci in NPC [19] and commonly used for classification also revealed that K4413-Mi EBV and K4123-Mi EBV are closer to B95-8 and more different from NPC-related EBVs (Figure 3B, C). Furthermore, both K4413-Mi EBV and K4123-Mi EBV lacked the 30 bp deletion at the carboxyl terminus and a specific amino acid substitution (Asp) at codon 335 with reference to Gly in B95-8 LMP1, a feature that was reportedly present in over 90% of EBVs found in NPC biopsies [20]. Moreover, K4413-Mi EBV and K4123-Mi EBV were evidently more distant from AG876, EBV isolated from African Burkitt’s lymphoma (Figures 2, 3A). Specific comparison of sequences in the EBNA3 region that had been used for classification of different EBV subtypes [21], similarly revealed K4413-Mi EBV and K4123-Mi EBV were more closely related to B95-8, a subtype 1 EBV (Figure 2). They were most distant from AG876, a subtype 2 EBV from the Western African case of Burkitt’s lymphoma.

Inclusion of the 2 most recently reported sequences of EBV genomes, Akata and Mutu, in our analysis of genomic sequence variations reveals the significant geographical distribution factor in addition to the factors of disease or tissue association. K4413-Mi EBV and K4123-Mi EBV are clearly more closely related to B95-8 strain and WT-EBV and more different from GD1, GD2 and HKNPC1 EBVs associated with NPC in the East Asia and Akata-EBV strain from a Japanese case of Burkitt’s lymphoma. They are clearly most different from the EBV of Western Africa case of Burkitt’s lymphoma. However, it is interesting to find that Mutu, EBV strain from a Kenya case of Burkitt’s lymphoma in the East Africa is more closely related to K4413-Mi EBV, K4123-Mi EBV, B95-8 and WT-EBV found in the North America (Figure 3A). More genome sequencing data of EBVs from different geographic regions in the world in the future could provide important information of the history or the route of EBV dissemination as well as its evolution. In this study, the geographical distribution factor in sequence variations among these EBVs can similarly be observed from the alignments of translated amino acid sequences of LMP1 and EBNA-1 genes among these EBVs of different origins (Figures 3B, C).

It may also be important to note that there were 10 heterogeneous SNPs found in the K4413-Mi EBV genome. There was also 1 heterogeneous SNPs found in the K4123-Mi EBV genome. The finding would suggest that there could be more than two EBV variants or quasi species within the K4413-Mi cell and K4123-Mi cell.

## Conclusions

We used the Illumina MiSeq sequencing platform, a NGS technology and convenient computational tools, CLC Genomics Workbench, to identify and extract EBV sequences from human B lymphocyte cell lines that underwent spontaneous immortalization promoted by infection of mycoplasma in culture. Genomes of EBVs identified in the 2 immortalized human B lymphocytes were constructed and compared with all the other EBV genomes available in GenBank. The study has demonstrated how viral agents in infected human cells could be rapidly identified, characterized and compared at the genomic level through genomic sequencing and effective computational analysis. The EBVs found in these human B lymphocytes are most closely related to B95-8 and WT-EBV. They are less similar to EBVs found in NPC and Burkitt’s lymphoma found in the East Asia. They are most different from EBV associated with the Western African Burkitt’s lymphoma based on whole genome sequences.

## Methods

### Sample preparation and short-read DNA sequencing

Two EBV-positive human B cell lines that underwent spontaneous immortalization promoted by infections of mycoplasma (namely K4413-Mi and K4123-Mi) were cultured as described [17]. Both of the B-cell lines were established from buffy coats of healthy blood donors obtained from Department of Transfusion Medicine, Warren Grant Magnuson Clinical Center, National Institutes of Health (Bethesda, MD). Personal information concerning these samples had been recorded in such a manner that subjects could not be identified, directly or through identifiers according to the HIPAA rules. Thus, the ethnic and geographic origins of the donors are not available. Genomic DNAs from these cell lines were extracted using the DNAse Blood and Tissue Kit (Qiagen). 50 ng DNA from each cell line was subjected to DNA library construction using the Nextera DNA Kit (Illumina) according to the manufacturer’s protocol. Briefly, DNA library was prepared through procedures that include tagmentation and 5- cycle PCR amplification. The DNA library has insert sizes ranging from 250 bp to 1000 bp. Two sequencing runs were conducted for each sample studied. The 1st run sequencing used Illumina Miseq reagent kit (300 cycles for the 2 × 150 bp pair-end sequencing). The 2nd run of sequencing used Illumina Miseq reagent kit V2 (500 cycles for the 2 × 250 bp pair-end sequencing). The reads that passed Illumina quality control filtering were used as raw data for further bioinformatics analysis.

### Taxonomic analysis of the short sequencing reads

The reference human database (hg19 human reference assembly) was downloaded from the University of California, Santa Cruz (UCSC), (http://hgdownload.cse.ucsc.edu/downloads.html#human); bacterial, fungal and viral databases were downloaded from National Center for Biotechnology Information (NCBI) (ftp://ftp.ncbi.nlm.nih.gov/genomes/Bacteria/, 04/27/2013 version; ftp://ftp.ncbi.nlm.nih.gov/genomes/Fungi/, 03/25/2013 version; ftp://ftp.ncbi.nlm.nih.gov/genomes/Viruses/, 05/22/2013 version). These 4 databases were uploaded to the CLC Genomics Workbench to build the local database. All sequencing reads were first aligned to the hg19 human database using the CLC Genomics Workbench with the default parameters (http://www.clcbio.com/, version 6.0.2). The matched human reads were removed from the raw data set. Likewise, the remaining reads were aligned to the locally built bacterial, fungal and viral databases sequentially using the CLC Genomics Workbench.

### Assembly of the K4413-Mi EBV and K4123-Mi EBV genomes

The total raw reads of the K4413-Mi or K4123-Mi cell line were aligned to the WT-EBV by using the CLC Genomics Workbench with the default parameters. The matched reads for each cell line were used for the EBV genome assembly.

### Identification of variations in the EBV genomic sequences from the K4413-Mi and K4123-Mi cell lines

Using WT-EBV as a reference, the single nucleotide variations (SNVs), inserts and deletions (indels) and multiple nucleotide variations (MNVs) were identified from the K4413-Mi and K4123-Mi cell lines. MiSeq reads by using the CLC Genomics Workbench with the default parameters.

### Comparative and phylogenetic analysis

A global comparison and visualization of WT-EBV against K4413-Mi EBV, K4123-Mi EBV, NA12878 EBV, B95-8, AG876, Akata, Mutu, GD1, GD2 and HKNPC1 was performed using the mVISTA program (http://genome.lbl.gov/vista/mvista/submit.shtml) with 100-bp moving window.

The global sequence alignments of the 11 EBV genomes and individual gene sequence alignments were analyzed using Kalign and Clustal Omega in EBI (http://www.ebi.ac.uk/Tools/msa/kalign/; http://www.ebi.ac.uk/Tools/msa/clustalo/) respectively. The alignments were used to generate phylogenetic trees using Molecular Evolutionary Genetics Analysis (MEGA) software, version 5.0 [22] with Neighbour-joining (NJ) algorithm.

### Nucleic sequence accession numbers

The full-length sequences of K4413-Mi EBV and K4123-Mi EBV were submitted to the GenBank database with the accession number KC440852 and KC440851.

## Competing interests

The authors declare that they have no competing interests.

## Authors’ contributions

HYL and SCL conceived and designed the experiments. ST and BJL performed cell culture study. TWL and HYL conducted genomic sequencing. HYL and GCH analyzed the sequencing data. SCL oversaw the progress of project and reviewed all the study data. HYL and SCL wrote the original draft of the manuscript. TWL, GCH, BJL and ST contributed to the discussion and writing of the final version of the manuscript. All authors reviewed and approved the final manuscript.

## Supplementary Material

Additional file 1: Table S1 Variants identified in K4413-Mi EBV in comparison with WT-EBV. Click here for file

Additional file 2: Table S2 Variants identified in K4123-Mi EBV in comparison with WT-EBV. Click here for file
